# Neuroendocrine tumor G1 in sphenoid sinuses: A case report

**DOI:** 10.1016/j.radcr.2025.06.090

**Published:** 2025-07-28

**Authors:** Seito Fukagawa, Shiho Asami, Masashi Sugasawa, Yasuhiro Ebihara, Mitsuhiko Nakahira, Yasutaka Baba

**Affiliations:** a Department of Diagnostic Radiology, Saitama Medical University International Medical Center, Saitama, Japan; b Department of Head and Neck Surgery, Saitama Medical University International Medical Center, Saitama, Japan

**Keywords:** Apparent diffusion coefficient (ADC), Computed tomography (CT), Magnetic resonance imaging (MRI), Neuroendocrine tumor (NET), Paranasal sinuses

## Abstract

Neuroendocrine neoplasms (NENs) commonly arise in the gastrointestinal tract, pancreas or lungs. Among NENs, there have been some reports of neuroendocrine carcinoma (NEC) occurring in the paranasal sinuses, but neuroendocrine tumors (NETs) of the paranasal sinuses are particularly uncommon, with only a few cases report available. Furthermore, there have been no comprehensive reports focusing on imaging findings. We herein report a case of paranasal sinus NET G1, along with a literature review of the imaging characteristics. The patient was a 64-year-old woman who was asymptomatic and had been incidentally diagnosed with a sphenoid sinus tumor. Computed tomography (CT) and magnetic resonance imaging (MRI) revealed a mass in the sphenoid sinus with heterogeneous contrast enhancement and destruction of the surrounding bones, including the sphenoid bone, clivus, and petrous bone. The mass had invaded the sella turcica and pushed the pituitary gland upward. The average and minimum apparent diffusion coefficient (ADC) values were 0.796 and 0.601 (× 10^-3^ mm^2^/s), respectively. A tumor biopsy was performed, leading to the diagnosis of NET G1. Surgical resection was deemed unfeasible, and the patient was treated with radiation therapy. No tumor progression or recurrence was observed over the follow-up period of 10 years. Previous studies of gastrointestinal NETs have reported an association between ADC values and tumor differentiation. This suggests that ADC values may be useful for predicting the tumor grade in paranasal sinus NETs.

## Introduction

Neuroendocrine tumors (NETs) are a diverse group of neoplasms that are characterized by varying clinical manifestations and growth patterns. NETs mainly occur in the lungs, pancreas, and digestive tract. Although neuroendocrine carcinoma is uncommon, it has been documented in cases of head and neck cancer [1]. Among the various types of NETs, NET G1, previously referred to as a typical carcinoid, is the rarest in the head and neck region, with the supraglottic larynx being the most frequent site of occurrence [2]. Reports of NET G1 originating in the paranasal sinuses are extremely limited [[3], [4], [5], [6], [7], [8], [9]], and the lack of detailed reports on imaging characteristics makes a preoperative diagnosis particularly challenging [3]. Currently, there is no standardized treatment protocol for these tumors, although surgery is generally considered the primary approach [10].

We herein report an unresectable NET G1 originating in the sphenoid sinus of a 64-year-old woman and the radiographic findings, including the apparent diffusion coefficient (ADC).

## Case presentation

A 64-year-old woman was referred for a further examination because of a paranasal sinus tumor. The patient had a history of hypertension and had undergone bilateral hip replacement surgery. She had also been diagnosed with severe mitral regurgitation, and a sinus tumor was incidentally detected on magnetic resonance imaging (MRI) performed during the preoperative examination. However, the patient did not exhibit any subjective symptoms at that time. Blood tests showed no elevation in tumor markers (carcinoembryonic antigen (CEA) and neuron specific enolase (NSE)), and hormone levels, including prolactin, adrenocorticotropic hormone, thyroid-stimulating hormone, free T3, and free T4, were all within normal limits. A physical examination revealed no neurological abnormalities or cervical lymphadenopathies.

**Fig. 1:**
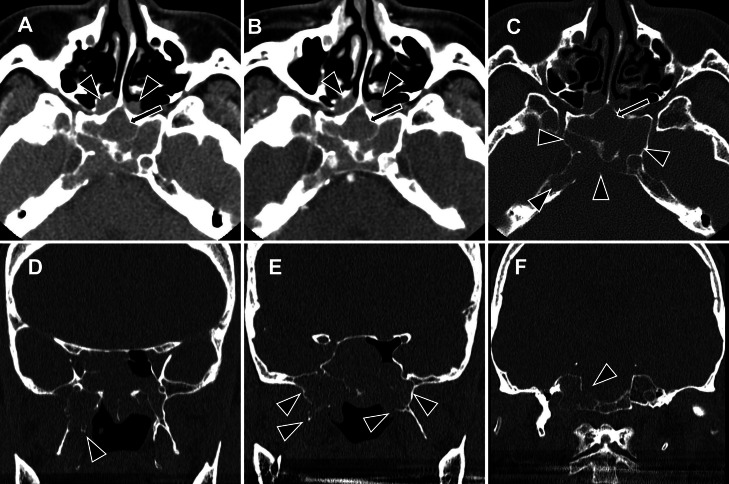
The mass in the sphenoid sinus on CT. (A) Noncontrast axial CT, (B) contrast-enhanced axial CT, (C) axial CT with bone window settings, and (D-F) coronal CT with bone window settings. A large mass is identified originating in the sphenoid sinus (A, B, arrows), with extension into the bilateral posterior ethmoid sinuses and nasal cavity (A, B, arrowheads). The tumor demonstrates heterogeneous enhancement following contrast administration (B). Widespread osteodestruction is evident. The sphenoid bone shows severe involvement, including the posterior aspects of the greater wings (right > left) (C, D, E) and the superior portions of the pterygoid plates (on the right, both plates; on the left, the medial plate only) (D, E). The lesser wings, however, are preserved. The destruction also involves the clivus and posterior occipital bone, with right-sided predominance (C, F).

**Fig. 2:**
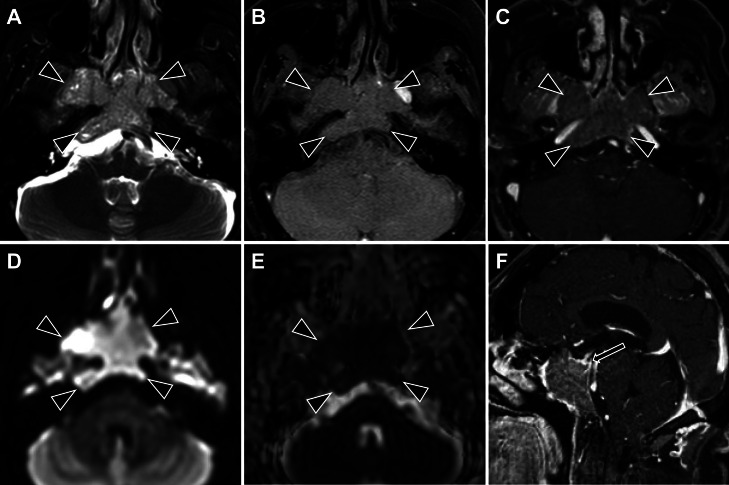
The mass in the sphenoid sinus on MRI. Axial images include a T2-weighted image (A), noncontrast T1-weighted image (B), postcontrast T1-weighted image (C), a diffusion-weighted image (D, b = 1000 s/mm²), and an ADC map (E). A sagittal postcontrast T1-weighted image is also shown (F). On imaging, the tumor demonstrates heterogeneous signal intensity on T2WI, composed of isointense and hyperintense signals relative to the skeletal muscles (A). The lesion is isointense on precontrast T1WI (B) and reveals weak, heterogeneous enhancement following gadolinium administration (C). Diffusion-weighted imaging reveals restricted diffusion, evidenced by high signal intensity (D) and low ADC values (mean: 0.796, min: 0.601 × 10⁻³ mm²/s). There is no evidence of intratumoral necrosis or cystic components. The tumor shows extensive invasion, extending into the right infratemporal fossa, destroying the clivus and dorsum sellae, and infiltrating the sella turcica and cavernous sinus (C, F). This results in superior displacement of the pituitary gland (F, arrow). The internal carotid artery remains patent without stenosis (C). Perineural invasion is not observed.

Pre- and postcontrast computed tomography (CT) (Fig. 1) and MRI (Fig. 2) revealed a mass that originated in the bilateral sphenoid sinuses and spread upward into the bilateral ethmoid sinuses, with a size of 56 × 39 × 32 mm. CT demonstrated a soft-tissue density mass that showed weak contrast enhancement. On MRI, the tumor was isointense on T1-weighted sequences and heterogeneous on T2-weighted sequences, containing a mixture of isointense and hyperintense signals. The tumor showed high signal intensity areas on diffusion-weighted images (DWI). The average and minimum apparent diffusion coefficient (ADC) values were 0.796 and 0.601 (× 10^-3^ mm^2^/s), respectively. Following gadolinium administration, it showed weak and heterogeneous enhancement. There was extensive osteodestruction. The greater wings of the sphenoid bone were largely destroyed, more so on the right, which corresponded to tumor extension into the right infratemporal fossa. The pterygoid processes were also involved; both plates were destroyed on the right, whereas only the superior portion of the medial plate was affected on the left. The walls of the sphenoid sinus were diffusely destroyed, though the lesser wings remained intact. The destruction extended to the clivus and posterior occipital bone, again with right-sided predominance. Intrasellar and cavernous sinus invasion was also present, resulting in superior displacement of the pituitary gland. Consequently, these findings suggested that the tumor originated in the sphenoid sinus. No calcification, cystic components, or necrosis was observed, and no lymph nodes were observed in the neck.

From these clinical and imaging findings, we considered several differential diagnoses, primarily squamous cell carcinoma and malignant lymphoma. We most strongly suspected squamous cell carcinoma, given the extensive bone destruction and its high frequency of occurrence in the paranasal sinuses. Malignant lymphoma was also a possibility due to the high signal on DWI and low ADC value; however, the extensive bone destruction was an atypical feature. The differential diagnosis also included other rare tumors, such as olfactory neuroblastoma, pheochromocytoma, melanoma, and sarcoma.

**Fig. 3:**
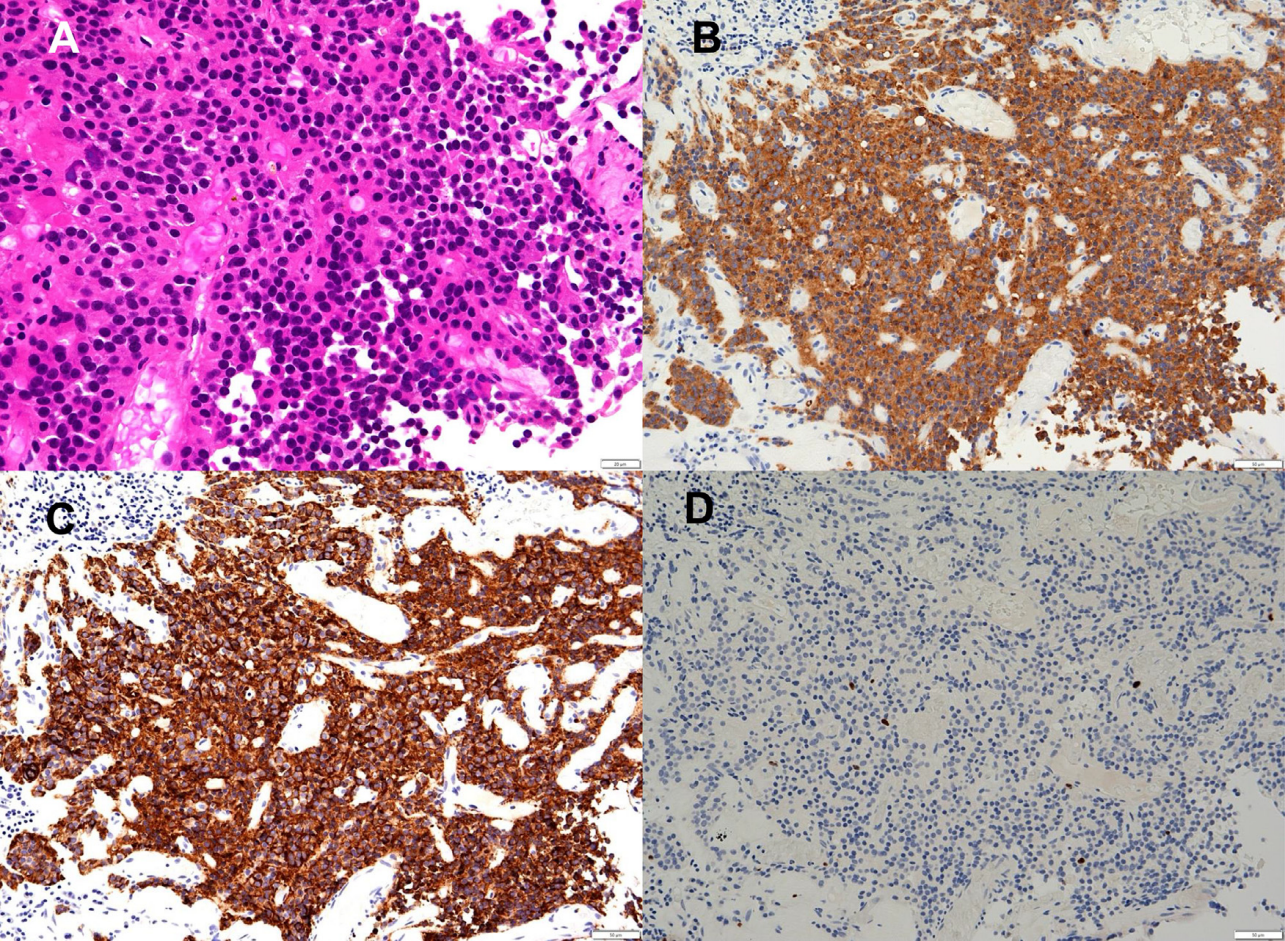
Pathology of the biopsied lesion. Hematoxylin and eosin staining show an alveolar proliferation of atypical cells with hyperchromatic, enlarged, round-to-oval nuclei (A). These cells are positive for synaptopysin (B) and chromogranin A (C). The Ki-67 proliferative index was <1% (D).

A biopsy of the lesion in the left nasal cavity was performed. Microscopic examination (Fig. 3) revealed tumor cells with oval to round nuclei and granular-looking chromatin, with nesting trabecular patterns around capillary vessels. The cells were relatively uniform, with no mitosis. Tumor cells were strongly and diffusely positive for synaptophysin (Syn), chromogranin A (CgA), and insulinoma-associated protein 1 (INSM1). The cells were negative for S-100, NKX2.2, and SSPI2. The Ki-67 proliferative index was <1%. The pathological diagnosis was neuroendocrine tumor (NET) G1 (also known as a typical carcinoid) of the sphenoid sinus.

18F-Fluorodeoxyglucose (FDG) positron emission tomography (PET)/CT, cystoscopy, and upper and lower gastrointestinal endoscopy were performed at another hospital to search for other tumors; however, no tumors were found outside the paranasal sinuses, and the tumor was thought to be primary in the paranasal sinuses.

The clinical staging of this tumor, based on the American Joint Committee on Cancer (AJCC) TNM staging system for the nasal cavity and paranasal sinuses [11], was T4bN0M0. As there was no indication for tumor resection, radiation therapy was administered. The patient was treated with stereotactic radiation therapy (SRT) and received a total dose of 35 Gy in 5 fractions.

After radiation therapy, CT showed a stable tumor with no evidence of metastatic disease after 10 years of follow-up.

## Discussion

NETs originate from specialized neuroendocrine cells. While rare, these tumors can develop anywhere in the body, with most cases occurring in the lungs, appendix, small intestine, rectum, and pancreas [12].

According to the 2022 WHO Classification of Endocrine and Neuroendocrine Tumors [13], well-differentiated epithelial neuroendocrine neoplasms are classified as NETs and graded as G1 to G3. Poorly-differentiated epithelial neuroendocrine neoplasms are classified as Neuroendocrine carcinomas (NECs), which are further categorized as small- or large-cell based on their cytomorphological features (Table 1).

**Table 1:** The 2022 WHO classification of neuroendocrine neoplasms.

		Pathological features
Neuroendocrine tumors (NETs)	G1	no necrosis and <2 mitoses per 2 mm^2^, Ki67 <20%
	G2	necrosis or 2-10 mitoses per 2 mm^2^, Ki67 <20%
	G3	>10 mitoses per 2 mm^2^ or Ki67 >20%, without poorly differentiated cytomorphology
Neuroendocrine carcinomas (NECs)	Small-cell or large-cell neuroendocrine carcinoma	>10 mitoses per 2 mm^2^, Ki67 >20%, and typically Ki67 >55%

In the head and neck region, G1 NETs most commonly develop in the middle ear and temporal bones. While NECs were previously thought to occur most frequently in the larynx, recent studies using updated classifications have identified the paranasal sinus as the predominant site. In summary, although NECs are relatively common in the paranasal sinuses, NETs in this region are exceptionally rare, with only sporadic cases documented in the literature [14].

A literature search revealed 7 previously reported cases of NET G1 originating in the paranasal sinuses [[3], [4], [5], [6], [7], [8], [9]]. The reported imaging findings shared several common characteristics. CT showed soft-tissue density masses with adjacent bone destruction and heterogeneous contrast enhancement. On MRI, the tumors were heterogeneous on both T1- and T2-weighted images, with signal intensities ranging from isointense to hyperintense relative to the brain parenchyma. Gadolinium-enhanced images showed heterogeneous enhancement, with some tumors containing cystic components. Consistent with these findings, our case also presented as a heterogeneous mass with widespread bone destruction.

As paranasal sinus NETs are uncommon and lack comprehensive imaging studies, findings from NETs in other organs may provide valuable insights. The clinical distinction between G1/G2 NETs, which typically follow an indolent course, and more aggressive G3 NETs is crucial. Studies on pancreatic and intestinal NETs have suggested that ADC values correlate with malignancy [15]. In our case, despite a relatively low average ADC of 0.796 (× 10^-3^ mm^2^/s), this parameter was unsuitable for malignancy assessment. To our knowledge, this is the first reported ADC measurement for paranasal sinus NETs.

Ectopic pituitary neuroendocrine tumors (PitNETs), which develop outside the sella turcica [16,17], were considered in the differential diagnosis, given the sphenoid sinus origin. While the sphenoid sinus is the most common site for ectopic PitNETs [18] and their cellular morphology can be indistinguishable from other NETs, several factors argue against this diagnosis in our case: normal hormone levels, absence of hormone production on tissue staining, and no cytological features suggesting a pituitary origin. While staining for PIT1, TPIT, and SF1 could have been diagnostic, the limited sample quantity precluded this analysis.

No standardized treatment protocol exists for paranasal sinus NETs. Treatment options include surgery or radiation therapy for nonfunctional PitNETs [19], with surgical resection typically preferred for other NETs [13]. In our case, given the unresectable nature of the tumor, radiation therapy was administered, which resulted in stable disease for 10 years. Previous case reports have documented successful radiation treatment for NETs, suggesting that this may be an effective therapeutic option.

## Conclusion

We herein report a rare case of NET G1 occurring in the paranasal sinuses. Studies on NETs in other sites, such as the pancreas, have suggested that the ADC value on MRI correlates with malignancy; however, this was not the case in the present patient. Ectopic PitNETs remain in the differential diagnosis as a possible origin of NETs and may be confirmed by special pathological staining.

## Patient consent

The written informed consent was obtained from the patient for the publication of this case report and accompanying images.
